# Effects of temperature on metabolic rate during metamorphosis in the alfalfa leafcutting bee

**DOI:** 10.1242/bio.060213

**Published:** 2023-12-29

**Authors:** Kayla N. Earls, Jacob B. Campbell, Joseph P. Rinehart, Kendra J. Greenlee

**Affiliations:** ^1^ Department of Biological Sciences, North Dakota State University, Fargo, ND 58108, USA; ^2^ Edward T. Schafer Agricultural Research Center, US Department of Agriculture/Agricultural Research Station, Fargo, ND 58102, USA

**Keywords:** Closed system respirometry, Oxygen consumption, Solitary bees, *Megachile rotundata*, Thermal performance curve

## Abstract

Spring conditions, especially in temperate regions, may fluctuate abruptly and drastically. Environmental variability can expose organisms to temperatures outside of their optimal thermal ranges. For ectotherms, sudden changes in temperature may cause short- and long-term physiological effects, including changes in respiration, morphology, and reproduction. Exposure to variable temperatures during active development, which is likely to occur for insects developing in spring, can cause detrimental effects. Using the alfalfa leafcutting bee, *Megachile rotundata*, we aimed to determine if oxygen consumption could be measured using a new system and to test the hypothesis that female and male *M. rotundata* have a thermal performance curve with a wide optimal range. Oxygen consumption of *M. rotundata* pupae was measured across a large range of temperatures (6–48°C) using an optical oxygen sensor in a closed respirometry system. Absolute and mass-specific metabolic rates were calculated and compared between bees that were extracted from their brood cells and those remaining in the brood cell to determine whether pupae could be accurately measured inside their brood cells. The metabolic response to temperature was non-linear, which is an assumption of a thermal performance curve; however, the predicted negative slope at higher temperatures was not observed. Despite sexual dimorphism in body mass, sex differences only occurred in mass-specific metabolic rates. Higher metabolic rates in males may be attributed to faster development times, which could explain why there were no differences in absolute metabolic rate measurements. Understanding the physiological and ecological effects of thermal environmental variability on *M. rotundata* will help to better predict their response to climate change.

## Teaser

**Summary:** Ectotherms can experience a wide range of optimal to lethal temperatures due to environmental variation. *Megachile rotundata* is a solitary bee that is immobile during spring development and must endure changes in temperature. Thermal performance curves were generated for developing *Megachile rotundata* pupae using a new respirometry system.

## INTRODUCTION

Temperatures experienced by organisms vary greatly, depending on location, season, and microhabitat. Large temperature fluctuations can have dramatic effects on ectotherms, such as insects, because of their reliance on ambient temperature to maintain body temperature. During the spring when temperatures are particularly variable and unpredictable, many insects are undergoing development and thus, may have to endure these temperature fluctuations (von Schmalensee et al., 2021). Even short bouts of unfavorable thermal conditions can have lasting effects on developing organisms (Earls et al., 2021; Jentsch et al., 2007; Sgrò et al., 2016; Zizzari and Ellers, 2011).

Depending on its microhabitat, an insect may experience varying thermal profiles, which can differ greatly from large-scale climate data (Sheldon and Dillon, 2016; Woods et al., 2015). Mobile insects can select more ideal microclimates or behaviorally thermoregulate (Cook et al., 2016; Heinrich, 1975; Heinrich and Esch, 1994; Heinrich and Vogt, 1993; Jones and Oldroyd, 2007; Kingsolver et al., 2011; Westhus et al., 2013). However, immobile insects, especially those undergoing metamorphosis inside a cocoon, are limited in their ability to avoid exposure to unfavorable temperatures (Kingsolver et al., 2011).

One way to evaluate thermal effects on insects is by generating a thermal performance curve (TPC) that shows the relationship of insect performance across a range of ambient temperatures (T_a_; Deutsch et al., 2008, Martin and Huey, 2008, Sinclair et al., 2012). Performance can be measured through various physiological and life history traits that are time-dependent (Schulte et al., 2011). The resulting curve is asymmetric and increases with increasing temperature up to a point. The range of T_a_ can be separated into four categories based on the response of the performance metric: suboptimal, optimal, supraoptimal, and lethal. Typically, the range of T_a_ surrounding the peak of the performance curve is considered optimal. The performance metric gradually decreases with T_a_ below the optimum, whereas above the optimum, the performance metric steeply decreases until T_a_ reaches CTmax or the upper lethal temperature (Blanckenhorn et al., 2021; Martin and Huey, 2008; Schulte et al., 2011). TPC can differ among species and with the performance metric used (e.g. locomotion or metabolic rate; Blanckenhorn et al., 2021; Martin and Huey, 2008; Schulte et al., 2011).

Many studies have used metabolic rate to generate a TPC in ectothermic species (Flynn and Todgham, 2018; Schulte et al., 2011; Shah et al., 2021; Tomlinson, 2019). Thermal performance curves can be limited in their ability to predict fitness in response to fluctuations in environmental temperature; however, they are a strong starting point for understanding the organismal response to thermal changes (Clarke, 1991; Sinclair et al., 2016). For immobile and non-feeding life stages, measuring metabolic rate is one of few temperature dependent reactions that can be measured. Understanding the effects of suboptimal T_a_ on metabolic rate is important for identifying how these insects respond to abrupt changes in environmental conditions.

The alfalfa leafcutting bee, *Megachile rotundata*, is an example of an insect that is restricted in its ability to seek more favorable thermal conditions during development. As a cavity-nesting bee species, offspring remain within brood cells where they were oviposited until adult emergence (Trostle and Torchio, 1994). Adult emergence may happen within the same season or the following year, depending on diapause status. Offspring that overwinter as prepupae are exposed to a long series of changes in temperatures as they are confined within their brood cells for 6–9 months spanning late summer, fall, winter, and spring (Krunic, 1972; Pankiw et al., 1980, Wilson et al., 2021; Yocum et al., 2018). In artificial nesting boxes, temperatures can range from over 45°C in the summer to below 0°C in the winter depending on location (Wilson et al., 2021; Yocum et al. 2006). During diapause, *M. rotundata* may have physiological protection from changing T_a_, such as metabolic suppression. However, once spring temperatures increase and active development is initiated, bees may be vulnerable to fluctuations in T_a_. *Megachile rotundata* is also an extensively managed solitary bee used for agricultural pollination from Canada and throughout the USA and, as a species, experiences a wide range of temperatures (Pitts-Singer and James, 2005, 2008). Previous research has mostly examined survival and delayed development of *M. rotundata* when pupae are subjected to low temperature stress with varying durations (Bennett et al., 2015; Earls et al., 2021; Kemp and Bosch, 2000; Rinehart et al., 2011; Underraga and Stephen, 1980b; Yocum et al., 2010, 2019). Despite numerous studies on the effects of temperature on *M. rotundata*, few have measured the direct effects of temperature organismal level as they are occurring.

Using oxygen consumption (V̇O_2_) as a proxy for metabolic rate as the performance metric across T_a_, our objectives were to (1) determine whether V̇O_2_ could be accurately measured while pupae remained in their brood cells; (2) generate a thermal performance curve (TPC) for developing *M. rotundata*; and (3) determine whether there were sex differences in the metabolic response to temperature in *M. rotundata* pupae. Our hypothesis was that V̇O_2_ in male and female *M. rotundata* pupae would exhibit a wide optimal temperature range. To test this hypothesis, we used a new multiplex technique for measuring V̇O_2_ and validated the measurement using traditional closed-system respirometry. Overwintering *M. rotundata* prepupae were allowed to develop into pupae at which time they were exposed to temperatures ranging from 6–48°C. To better characterize the metabolic profile of *M. rotundata*, multiple comparisons of V̇O_2_ were made, including between sexes, pupae remaining inside their brood cells and those extracted, between seasons, and absolute and mass-specific metabolic rates. Measuring responses, such as metabolic rate, to T_a_ during development can be used to understand how the effects of small-scale changes affect physiological processes that can have long-term effects.

## RESULTS

### Comparison of closed respirometry systems

**Fig. 1.**
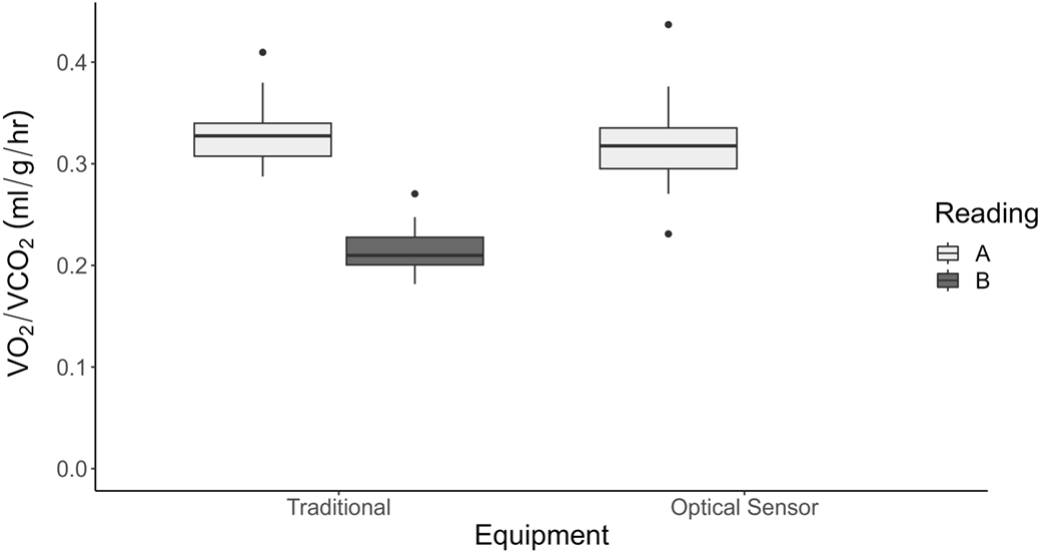
**Comparison of mass-specific metabolic rate calculations from both respirometry systems.** Oxygen consumption was not statistically different between systems (two-tailed *t*-test; t=−0.682, d.f.=25, *P*=0.502; *n*=20 bees per system). Carbon dioxide was measured concurrently with oxygen in the fuel cell system but not in the microplate system.

There was no statistical difference in V̇O_2_ between pupae measured in the microplate system (0.319±0.012 ml/g/h) and the fuel cell analyzer (0.331±0.011 ml/g/h) at 20°C (t=−0.682, d.f.=25, *P*=0.502). V̇CO_2_ (0.215±0.008 ml/g/h) was recorded simultaneously with V̇O_2_ when we used closed system respirometry and the fuel cell analyzer (Fig. 1). The calculated respiratory quotient (RQ=CO_2_/O_2_) was 0.651±0.006.

### Objective 1

**Fig. 2.**
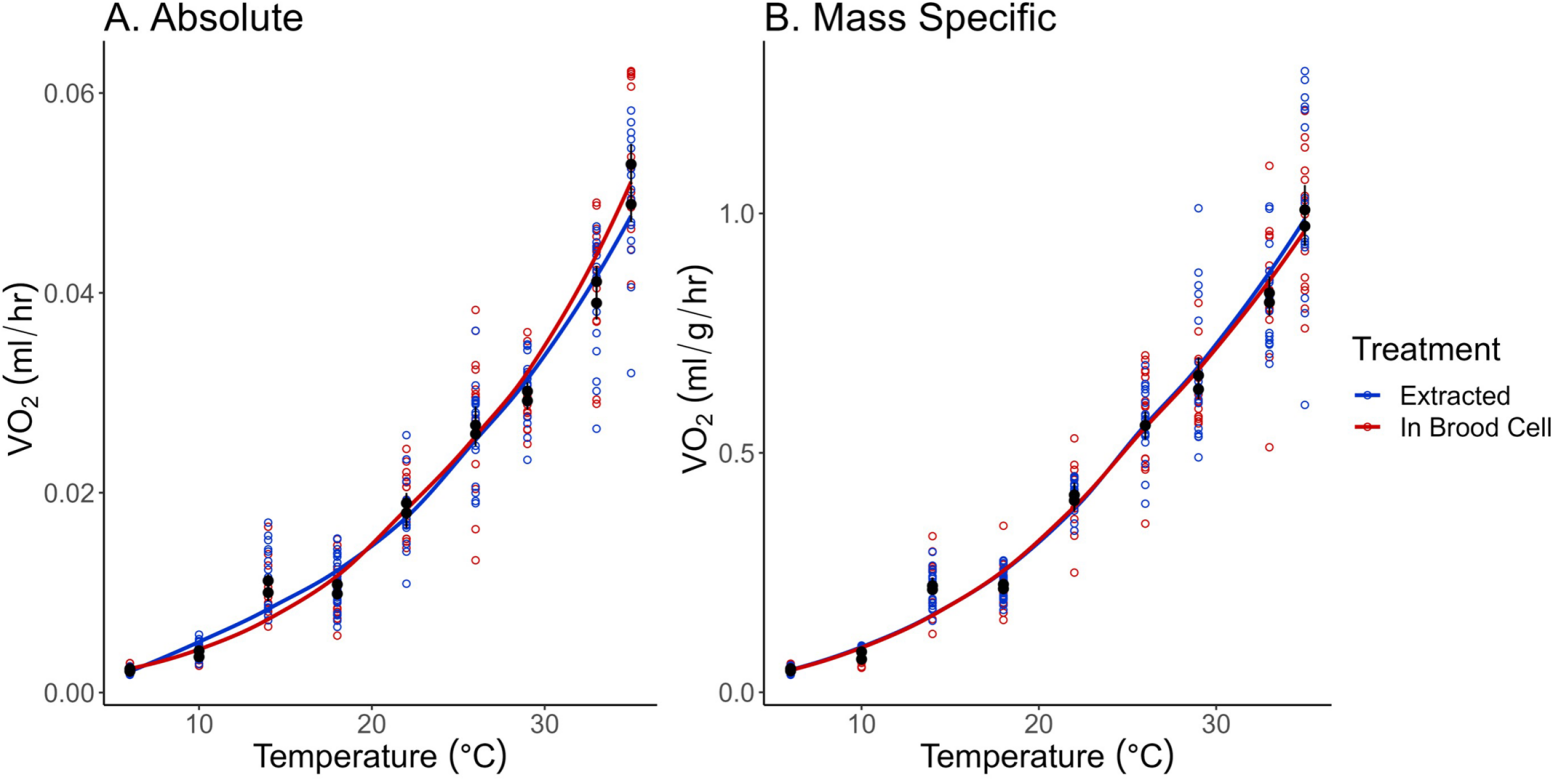
**V̇O_2_ for bees left in their brood cells (red triangles) and those that were extracted from their brood cells (blue circles) during measurements in objective 1**. No statistical differences (*P*>0.05) were detected by two-way ANOVA in absolute (A) and mass-specific (B) metabolic rates between bees that were extracted (*n*=16) or left in their brood cells (*n*=16). Open colored circles indicate individual bee measurements, while the black circles and triangles represent the mean±s.e.m.

*Megachile rotundata* that remained in the brood cell and those that were extracted for measurements did not differ in either absolute (*F*_1,290_=0.423, *P*=0.516) or mass-specific (*F*_1,290_=1.29, *P*=0.258) V̇CO_2_; therefore, the treatments were combined for further analysis. Absolute and mass-specific V̇O_2_ varied significantly across T_a_ (absolute: *F*_8,290_=909, *P*<0.0001, Fig. 2A; mass-specific: *F*_8,290_=1264, *P*<0.0001, Fig. 2B). Metabolic rate increased non-linearly as temperatures increased and were best fit by a third degree polynomial curve (Table 1).

**Table 1.**
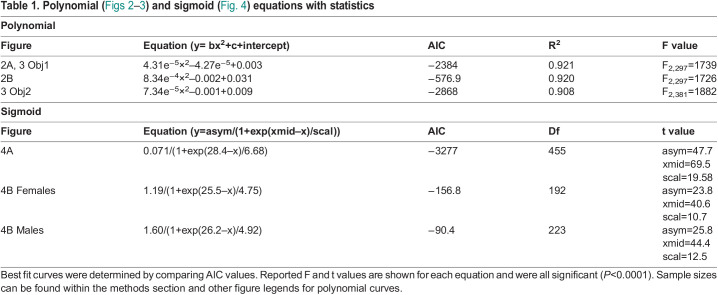
**Polynomial (**Figs 2–3**) and sigmoid (**Fig. 4**) equations with statistics**

**Sigmoid**				
**Figure**	**Equation (y=asym/(1+exp(xmid–x)/scal))**	**AIC**	**Df**	**t value**
4A	0.071/(1+exp(28.4–x)/6.68)	−3277	455	asym=47.7 xmid=69.5 scal=19.58
4B Females	1.19/(1+exp(25.5–x)/4.75)	−156.8	192	asym=23.8 xmid=40.6 scal=10.7
4B Males	1.60/(1+exp(26.2–x)/4.92)	−90.4	223	asym=25.8 xmid=44.4 scal=12.5

**Fig. 3.**
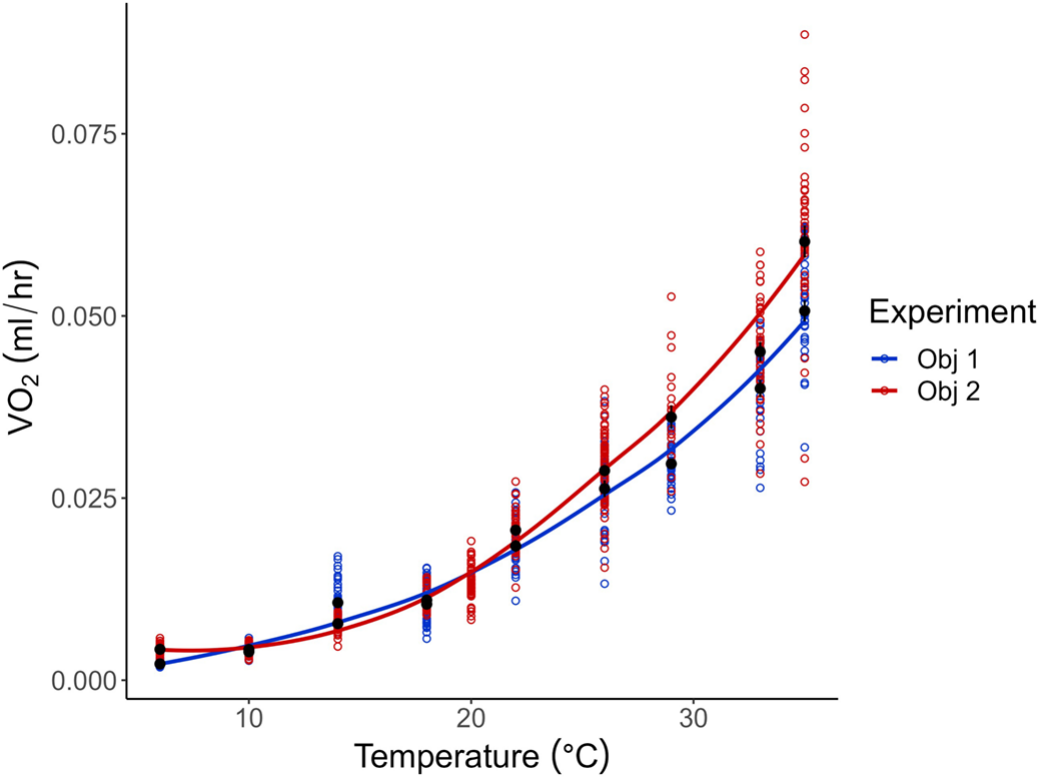
**Absolute V̇O_2_ between the first (*n*=32 per temperature) and second (*n*=40 per temperature) objectives.** There was a significant difference in absolute V̇O_2_ between objectives 1 and 2 (two-way ANOVA, *F*_1,673_=49.3, *P*<0.0001). Additionally, pupae used for objective 1 weighed 4 mg more than those used for objective 2 (objective 1: 0.0487±0.0005 g; objective 2: 0.0444±0.0004 g). Open colored circles indicate individual bee measurements, while the black circles and triangles represent the mean±s.e.m.

**Fig. 4.**
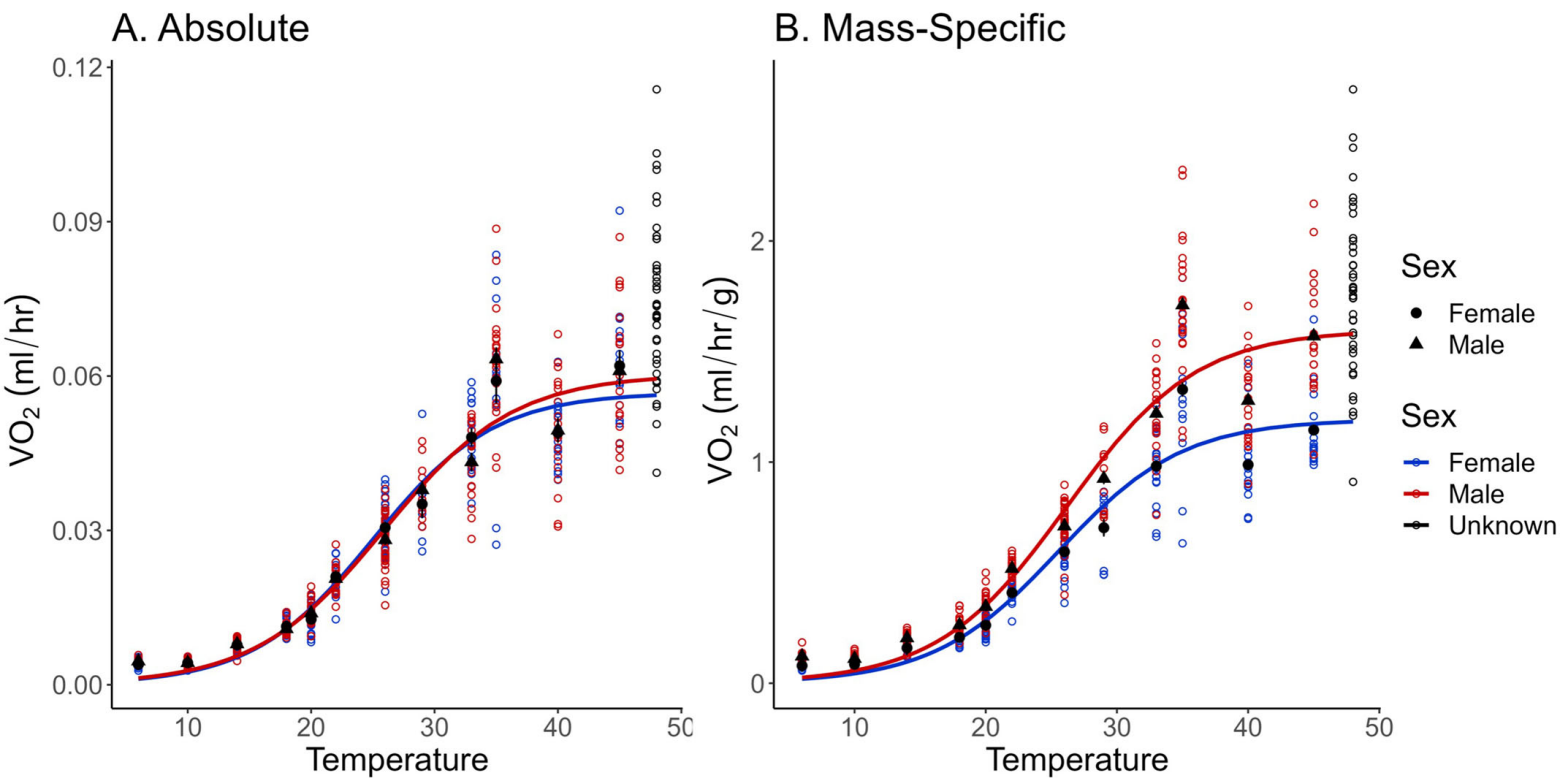
**V̇O_2_ for female and male bees measured by the optical oxygen sensor system in objective 2**. Pupae at 48°C were unable to complete development are represented by black open circles, because we were unable to determine sex. Those bees are not included in the regression. There was a significant difference in both absolute (A; two-way ANOVA, *F*_11,445_=1160, *P*<0.0001) and mass-specific (B; two-way ANOVA, *F*_1,434_=239, *P*<0.0001) V̇O_2_ across temperatures. (A) No statistical differences (*F*_1,445_=0.0464, *P*=0.830) were detected between absolute V̇O_2_ of female and male pupae across T_a_ (*n*=40 bees per temperature). (B) Mass-specific V̇O_2_ between sexes was significantly different across temperatures (*n*=40 bees per temperature). The number of males and females differed across temperatures (see Materials and Methods). Open colored circles indicate individual bee measurements, while the black circles and triangles represent the mean±s.e.m.

### Objective 2

**Fig. 5.**
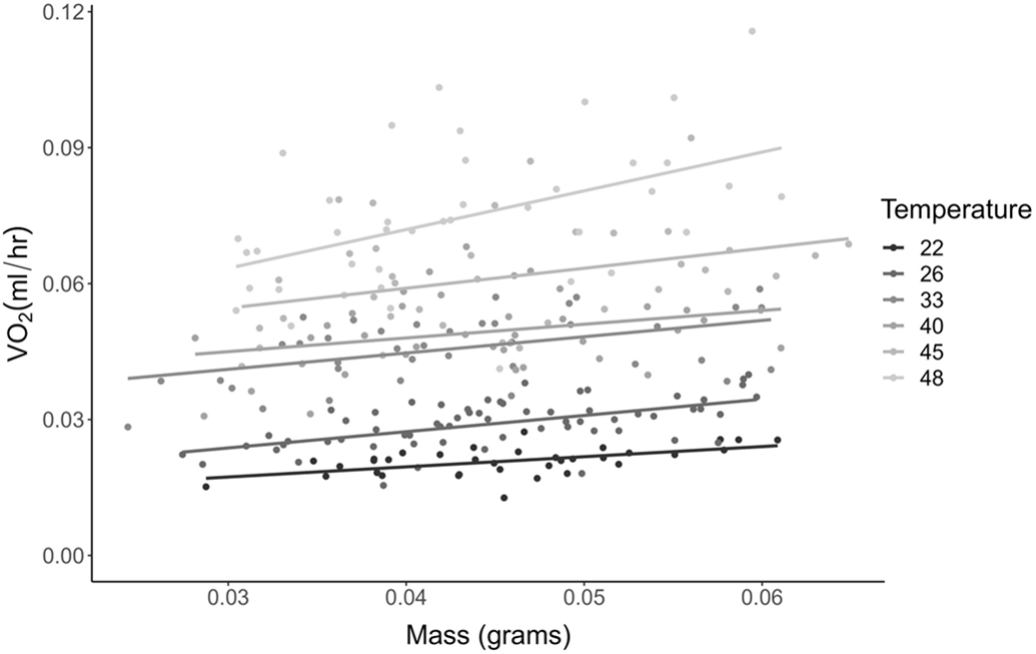
**Six of the temperature treatments resulted in significant regressions of oxygen consumption versus mass.** Individual points represent a single *M. rotundata* pupa (*n*=40 per temperature treatment). Individual regressions were performed on all the temperatures to determine which are significant (*P*<0.05). An ANCOVA was performed on the significant regressions (*F*_5,244_=255, *P*<0.0001). All slopes differed significantly from each other except for 33°C and 40°C.

None of the bees measured at 48°C finished development and, thus, were excluded from the analysis and figures, because sex can only be confidently determined at the adult stage. Q_10_ values calculated using absolute metabolic rate decreased by half at 29°C and continued to gradually decrease (Table 2). Absolute metabolic rate increased across T_a_ before plateauing (*F*_11,445_=1160, *P*<0.0001), but did not differ between sexes (*F*_1,445_=0.0464, *P*=0.830; Fig. 4A). However, mass-specific metabolic rate differed between sexes across T_a_ with males having higher oxygen consumption than females (*F*_1,434_=239, *P*<0.0001; Fig. 4B). Males weighed (0.0390±0.0004 g) less than female bees (0.0505±0.0004 g; t=19.808, d.f.=456, *P*<0.0001). All slopes generated in this objective were best fit by a sigmoid logistic regression (Table 1). A positive relationship between absolute V̇O_2_ and mass was determined in the following temperatures: 22°C (*F*_1,33_=11.7, *P*=0.0017, R^2^=0.239), 26°C (*F*_1,62_=12.0, *P*<0.0001, R^2^=0.149), 33°C (*F*_1,36_=13.3, *P*<0.0001, R^2^=0.250), 40°C (*F*_1,35_=5.72, *P*=0.022, R^2^=0.116), 45°C (*F*_1,35_=6.11, *P*=0.018, R^2^=0.124), and 48°C (*F*_1,38_=10.2, *P*=0.0029, R^2^=0.19; Fig. 5). Based on the Tukey *post-hoc* tests, all six slopes were significantly different from each other except for 33°C and 40°C (*F*_12,491_=797, *P*<0.0001; Fig. 5).

**Table 2.**
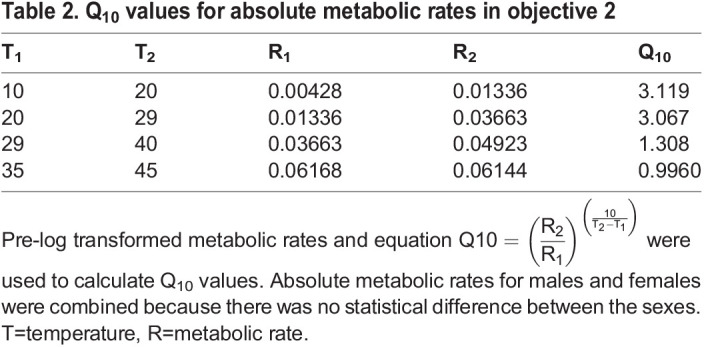
Q_10_ values for absolute metabolic rates in objective 2

Generally, objective 2 bees had higher oxygen consumption than bees used in objective 1 (*F*_1,673_=49.3, *P*<0.0001; Fig. 3). Pupae in objective 1 weighed 4 mg more than pupae in objective 2, (t=6.55, d.f.=682, *P*<0.0001). The average mass for bees in objective 1 was 0.0487±0.0005 g and from objective 2, the average mass was 0.0444±0.0004 g.

## DISCUSSION

Ectotherms, such as insects, rely on ambient temperature for body temperature. Previous research looking at development of *M. rotundata* has shown that temperature affects development rate, lipid content, adult flight performance, and reproduction (Bennett et al., 2015; Earls et al., 2021; O'Neill et al., 2011; Yocum et al., 2010). Performance traits, such as metabolic rate, respond non-linearly to temperature, resulting in an asymmetric thermal performance curve (Blanckenhorn et al., 2021; Martin and Huey, 2008; Schulte et al., 2011). The purpose of this study was to determine how V̇O_2_ of *M. rotundata* pupae responds to changes in temperature. Firstly, our prediction that V̇O_2_ would not differ between bees left in their brood cells versus being extracted out was met. The optical oxygen sensor was sensitive enough to measure V̇O_2_ while pupae remained in their brood cells. Developing *M. rotundata* are highly susceptible to handling stress and injury. Being able to measure gas exchange in this life stage while in their brood cell opens many possibilities for future experiments in this and other sensitive insects. Also, our results show that brood cells are highly permeable to oxygen.

Our hypothesis that V̇O_2_ responds to temperature variation in a traditional TPC was partially supported. Non-linear slopes of the relationship between metabolic rate and T_a_ were observed in both objectives and sexes; however, the predicted negative slope at T_a_ above the optimal range was not observed. One explanation is that the exposure time (≤2 h) may have been too brief to see the predicted negative slope and to capture thermal damage at supraoptimal T_a_. Another, less likely, possibility is that we did not expose bees to high enough temperatures. Even though there was not a decrease in oxygen consumption, other physiological processes could be negatively affected. For instance, in *Manduca sexta* food consumption and growth rate decreased while respiration rate did not increase (Kingsolver and Woods, 1997). In *M. rotundata*, only 1 h at 40°C is enough to increase heat shock protein expression (HSP70 and HSP90; Yocum et al., 2005). Furthermore, none of the pupae in this study measured at 48°C completed melanization, even though individuals showed high levels of oxygen consumption while in the optical oxygen sensor system. Measuring only the respiratory response to stress as the metric for performance may be misleading, as the insect may be showing other physiological responses well before metabolic rate decreases (Kingsolver and Woods, 1997; Shah et al., 2021).

Although our exposures were short (≤2 h), even small increases in temperatures above optimal can have lasting effects. For instance, Asian lady beetles (*Harmonia axyridis*) that experienced high temperatures for 1 h as eggs had shorter development times, lower adult survival, and fewer offspring (Zhang et al., 2014). Short exposures of 2–4 h to high temperatures (40–50°C) negatively affected survivorship and reproduction of pupae in many different species, including the almond moth (*Ephestia cautella*), Indian meal moth (*Plodia interpunctella*), red flour beetle (*Tribolium castaneum*), and tobacco hornworm (*Manduca sexta*) (Arbogast, 1981; Kingsolver et al., 2011; Mahroof et al., 2005). At the adult stage, high temperature exposure can cause male sterility in *Drosophila buzzatii* and reduced fecundity in *Drosophila melanogaster* (Jørgensen et al., 2006; Krebs and Loeschcke, 1994). The effects of heat stress can also vary depending on life stage exposure. Negative effects on reproduction become more dramatic in later stages of development in the diamondback moth (*Plutella xylostella*) and the rose grain aphid (*Metopolophium dirhodum*) when measuring across life stages (Ma et al., 2004). Supraoptimal and suboptimal temperature ranges can both have long lasting effects on insect physiology.

Due to the asymmetric shape of a TPC, insects have a larger range of low temperatures that may not be lethal compared to the small range of high temperatures. However, during exposures to suboptimal temperatures, there can be long lasting effects on morphology and multiple physiological systems, including reproductive, muscular, and nervous systems (Earls et al., 2021; Hegdekar, 1971; Huang et al. 2007; Hutchinson and Bale, 1994; Kelty et al., 1996; Marshall and Sinclair, 2009; Rinehart et al., 2000; Turnock et al., 1983; Yocum et al., 1994). Insects that are exposed to low temperatures and then returned to optimal temperatures have been found to have higher metabolic rates than individuals that did not experience low temperatures (Lalouette et al., 2011; Williams et al., 2016), possibly indicating a physiological recovery response (Lalouette et al., 2011; Colinet et al., 2018). This could be tested by measuring post-exposure V̇O_2_ in *M. rotundata* and other insects with the optical oxygen sensor.

Our results showed that males had the same absolute V̇O_2_ compared to females (Fig. 3A), which was unexpected because males are smaller than females. Typically, males are oviposited last, making them closest to the entrance of the nest. As a result, males have to emerge as adults before females in the brood cells behind them, giving males shorter development times (Pitts-Singer and Cane, 2011). Previous research suggests that males and females have the same developmental threshold (18°C; Kemp and Bosch, 2000; O'Neill et al., 2011). Perhaps the higher than expected absolute V̇O_2_ is an indicator of the faster development times compared to females as explained by the pace of life hypothesis (Arnqvist et al., 2017). Pace of life hypothesis suggests that ‘living fast’ or using physiological resources faster results in short lifespans (Sandmeier and Tracy, 2014).

Clearly, changes in temperature can affect physiological and behavioral traits, such as reproduction, development times, foraging, and dispersal (Andrew et al., 2013). Previous research has shown that *M. rotundata* pupae are sensitive to large changes in temperatures that result in increased mortality, deformities, flight performance, and changes in reproductive investment (Bennett et al., 2015; Earls et al., 2021; Kemp and Bosch, 2000; Rinehart et al., 2011; Underraga and Stephen, 1980a, 1980b; Yocum et al., 2010, 2019). Constant temperatures were used for short periods of time which may mimic an acute thermal stress. However, the impacts of fluctuating temperatures on metabolic rate could be essential in understanding natural conditions and could be used in predictive models for insect responses to climate change (Faye et al., 2017; Maino et al., 2016; Sheldon and Dillon, 2016). Results from this study show that V̇O_2_ changed non-linearly across T_a_ and that males had higher mass-specific V̇O_2_ compared to females. Future studies could include the TPC generated in this study to determine the likelihood of occurrence and mechanisms contributing to negative effects of temperature stress. Additionally, the majority of bee species are solitary, therefore, *M. rotundata* can provide a beginning insight on how solitary bees respond to a wide range of temperatures during development. In conclusion, the TPCs from in this study can be used to create predictive models of the response to climate change and to better understand the physiological and ecological effects of thermal stress.

## MATERIALS AND METHODS

### Animal rearing

Loose brood cells containing post-diapause quiescent *Megachile rotundata* prepupae were purchased from JWM Leafcutters (Nampa, ID, USA) in March of 2020. Brood cells were stored in an incubator (Percival, Perry, IA, USA) at 6°C in complete darkness until use in the experiment. Bee development was initiated by placing bees in a 29°C incubator in a 30 ml plastic container with a fine mesh lid. Bees were allowed to develop for 2 weeks, at which time they reached the red-eye stage, a stage characterized by the darkened eye pigment (Bennett et al., 2015). A subset of bees was extracted prior to being exposed to low temperatures to ensure that they were at the correct pupal stage.

### Optical oxygen sensor

We measured oxygen consumption using closed-system respirometry with an optical oxygen sensor (Loligo Microplate System®, Viborg, Denmark). This respirometry system uses glass, 24-well microplates with individual optical fluorescence oxygen sensors attached to each gas-tight well (940 µl). The resolution of each sensor was±0.4% O_2_ at 20.9% O_2_. Up to nine plates can be used at once, allowing for even greater sample sizes. For all of the objectives, we used two microplates that were allowed to equilibrate overnight to the desired temperature. A two-point calibration using normoxia (room air) and anoxia (100% N_2_) was conducted at each temperature. To zero the optical sensors, wells were individually flushed with N4.8 grade N_2_ for 5 min at ∼41 ml/min. Nitrogen was pushed at a rate of 2000 ml/min with a mass flow meter (Sierra SideTrak840, Monterey, CA, USA) and mass flow controller (Sable Systems MFC-4, Las Vegas, NV, USA) through a polypropylene adaptor that we designed and milled with a CNC (computerized numerical control) machine from fused polypropylene blocks (Fig. S1). The adaptor split the flow of N_2_ to each of the wells individually, yielding a flow rate of ∼41 ml/min/well, assuming no leakage. To ensure the calibration gas was of the same temperature as the incubator, gases were pushed through copper tubing submerged in a water bath inside the incubator set at the incubator temperature. For the span gas (room air), wells were left open for at least 2 min after percent air saturation returned to 100% before the measurement. To verify that the Loligo Microplate System® was reading the correct amount of oxygen, wells were flushed with gas mixes of 21% and 18% O_2_ balance N_2_ at 25°C. Nitrogen and oxygen tanks were used to mix gases using the MFC-4, and percent air saturation was recorded.

### Objective 1 experimental design

Objective 1 bees were oviposited by females in summer 2019 and stored at 6°C for approximately 12 months until fall 2020. Red-eye pupae in brood cells (*n*=32 per temperature) were haphazardly chosen and removed from the 29°C incubator. Half of the bees (*n*=16) were placed in the wells in their brood cells, while the other half were extracted out of their brood cells and were evenly distributed between the two microplate wells. Eight wells on each plate were left empty to act as controls. Percent air saturation in each well was recorded every 30 s at the following temperatures: 6, 10, 14, 18, 22, 26, 29, 33, and 35°C, for 2 h or until oxygen levels reached 18%, a level well above the critical PO_2_ for *M. rotundata* (Owings et al., 2014). Bees were only measured at one temperature and weighed immediately post-exposure and placed in 29°C in humidity-controlled chambers to finish development so that we could determine the sex of each individual.

### Objective 2 experimental design

The experiment was replicated in spring 2021 with more temperatures and younger bees that were oviposited in summer 2020 and stored at 6°C for approximately 9 months only until spring of the following year. For objective 2, red-eye pupae were left in their brood cells during the measurement and the number of controls was reduced to four. Brood cells were haphazardly selected and then randomly assigned to wells by MicroResp™ (Loligo Systems, Viborg, Denmark). Percent air saturation in each well was recorded every 30 s at the following temperatures: 6, 10, 14, 18, 20, 22, 26, 29, 33, 35, 40, 45, 48°C (*n*=40 bees per temperature). Bees were only measured at one temperature and were weighed immediately post exposure and replaced in 29°C to finish developing in humidity-controlled chambers. Sex was determined when bees emerged as adults.

### Calculation of oxygen consumption

For both objectives 1 and 2, V̇O_2_ was calculated with the following equations:

absolute:

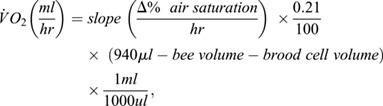
mass specific:

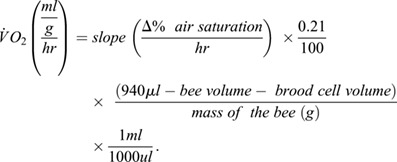
The slope was calculated using MicroResp™ software (Loligo Systems, Viborg, Denmark) using T_0_ as 10 min after the wells were sealed until the averaged percent oxygen reached 18% to reduce the effects of oxygen limitation. T_0_ was selected to reduce effects of opening and closing the incubator door on the percent oxygen saturation readings. Q_10_ was calculated comparing absolute V̇O_2_ from 10–20°C, 20–29°C, 29–40°C, and 35–45°C (Neven, 2000).

### Comparison to closed-system respirometry

To test the precision of the optical sensors, 20 bees were measured using the oxygen sensor system and a traditional closed respirometry system. Bees were reared to the red-eye stage as described above and left in their brood cells for the measurements. Measurements using the optical sensor system were performed as described above. For the traditional closed respirometry system, we injected samples into an airstream that was directed through a carbon dioxide analyzer (Li-Cor-7000, LI-COR Biosciences, Lincoln, NE, USA) and an oxygen analyzer (Oxzilla FC-2 Differential Oxygen Analyzer, Sable Systems International, Las Vegas, NV, USA) as previously done (Wang et al., 2016). Brood cells were individually placed into gas-tight 3-ml syringes fitted with two-way valves. Syringes were flushed with dry, CO_2_-free air (Balston Puregas system, Haverhill, MA, USA) for ∼30 secs at ∼200 ml min^−1^, ensuring that the starting levels of CO_2_ in the syringe were zero. Syringes were quickly sealed, placed into an incubator at 20°C, and left undisturbed for up to nine hours to ensure O_2_ depletion was detectable with the fuel cell oxygen analyzer. At the end of the incubation, the syringe containing the bee was connected to the respirometry system, and a bolus of 1 ml of air from the syringe was injected into the stream of the carrier gas (dry, CO_2_-free air) and pushed through the system at 100 ml min^−1^. Drierite was used to scrub water from the sample before it entered the CO_2_ analyzer, and a combination of Drierite and Ascarite was used to scrub water and CO_2_ before the sample entered the O_2_ analyzer. Peaks generated by the bolus injection were integrated and converted into ml of CO_2_ produced and O_2_ consumed as described in Lighton (2008), and then converted to ml g^−1^ hr^1^ by dividing by the time in the sealed chamber and the wet mass of the individual. As a control, empty syringes were periodically flushed with dry CO_2_-free air and injected into the respirometry system to ensure that flushing time and flow rate were sufficient to retain starting CO_2_ at zero and syringes were airtight. Pupal bees in the traditional closed respirometry system remained in the syringes for longer periods (∼9 h) than bees tested in the microplate system (∼3 h) due to the differences in chamber size (3 ml syringes versus 940 µl wells) and detection limits of the oxygen analyzers.

### Statistical analysis

Statistical analyses were performed in JMP Pro (version 17.0.0) and in R (version 1.1.423) (R Core Team, 2019) using *dplyr* for workflow, *car* for unbalanced designs and *nls2* to create sigmoid curves. Prepupae that died (indicated by no adult emergence) and pupae that were parasitized (indicated by the presence of parasitoid larvae during the pupal stage) were excluded from all analyses. ANOVA was used to test for effects of temperature, sex, brood cell status, and their interactions on metabolic rate. Sex, objective, and brood cell status were treated as covariates. Although data are presented in figures as linear, oxygen consumption was log_10_ transformed for statistical analysis to meet the assumptions of ANOVA. Curves were compared using AIC values to determine the best fit. In objective 2, linear regressions were performed for each temperature to compare metabolic scaling relationships. Significant regressions were then compared using ANCOVA. *t*-tests were used to compare differences in V̇O_2_ between the microplate and fuel cell oxygen analyzer, bee masses between males and females, and bee masses between objectives 1 and 2. Tukey *post-hoc* comparisons were used to detect significant differences across temperatures and groups. All data are presented as mean±standard error of the mean (s.e.m.), and significance was determined by *P*<0.05.

## Supplementary Material

10.1242/biolopen.060213_sup1 Supplementary information
