# Supplementary material for: Effects of temperature on metabolic rate during metamorphosis in the alfalfa leafcutting bee
Source: Biol Open. 2023 Dec 29;12(12):bio060213. doi: 10.1242/bio.060213 (PMC10805150; doi:10.1242/bio.060213)
Supplement: Supplementary information [file biolopen-12-060213-s1.pdf]

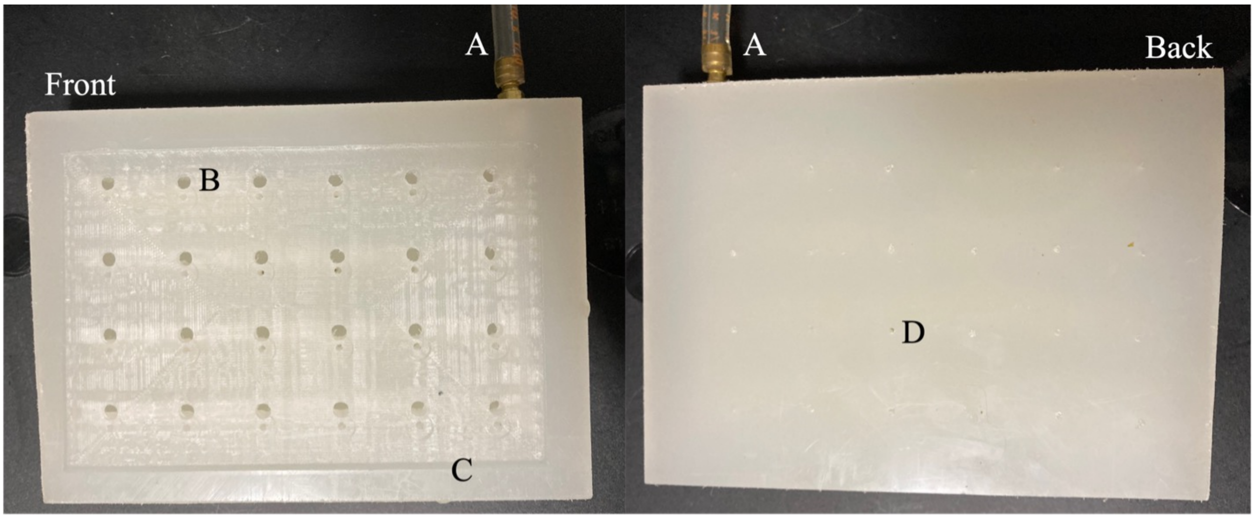

Fig. S1. Front and back views of the polypropylene adaptor was designed and milled with a CNC (computerized numerical control) from fused polypropylene blocks. For calibration N_2 flows through tubing **(A)** into the polypropylene block where the N_2 is delivered directly into each well through holes **(B).** Flushed N_2 leaves the polypropylene block through smaller holes **(D).** A ridge **(C)** was created to fit the adapter onto the glass well plates and prevent sliding.
